# Preparation of Electrodes with β-Nickel Hydroxide/CVD-Graphene/3D-Nickel Foam Composite Structures to Enhance the Capacitance Characteristics of Supercapacitors

**DOI:** 10.3390/ma17010023

**Published:** 2023-12-20

**Authors:** Yang-Ming Lu, Sheng-Huai Hong

**Affiliations:** Department of Electrical Engineering, National University of Tainan, Tainan 7005, Taiwan

**Keywords:** graphene, 3D nickel form, CVD, Capacitance, nickel hydroxide

## Abstract

Supercapacitors have the characteristics of high power density, long cycle life, and fast charge and discharge rates, making them promising alternatives to traditional capacitors and batteries. The use of transition-metal compounds as electrode materials for supercapacitors has been a compelling research topic in recent years because their use can effectively enhance the electrical performance of supercapacitors. The current research on capacitor electrode materials can mainly be divided into the following three categories: carbon-based materials, metal oxides, and conductive polymers. Nickel hydroxide (Ni(OH)_2_) is a potential electrode material for use in supercapacitors. Depending on the preparation conditions, two crystal phases of nickel hydroxide, α and β, can be produced. When compared to α-NiOH, the structure of β-Ni(OH)_2_ does not experience ion intercalation. As a result, the carrier transmission rate of α-Ni(OH)_2_ is slower, and its specific capacitance value is smaller. Its carrier transport rate can be improved by adding conductive materials, such as graphene. β-Ni(OH)_2_ was chosen as an electrode material for a supercapacitor in this study. Homemade low-pressure chemical vapor deposition graphene (LPCVD-Graphene) conductive material was introduced to modify β-Ni(OH)_2_ in order to increase its carrier transport rate. The LPCVD method was used to grow high-quality graphene films on three-dimensional (3D) nickel foam substrates. Then, a hydrothermal synthesis method was used to grow β-Ni(OH)_2_ nanostructures on the 3D graphene/nickel foam substrate. In order to improve the electrical properties of the composite structure, a high-quality graphene layer was incorporated between the nickel hydroxide and the 3D nickel foam substrate. The effect of the conductive graphene layer on the growth of β-Ni(OH)_2_, as well as its electrical properties and electrochemical performance, was studied. When this β-Ni(OH)_2_/CVD-Graphene/3D-NF (nickel foam) material was used as the working electrodes of the supercapacitor under a current density of 1 A/g and 3 A/g, they exhibited a specific capacitance of 2015 F/g and 1218.9 F/g, respectively. This capacitance value is 2.62 times higher than that of the structure without modification with a graphene layer. The capacitance value remains at 99.2% even after 1000 consecutive charge and discharge cycles at a current density of 20 A/g. This value also improved compared to the structure without graphene layer modification (94.7%).

## 1. Introduction

As fossil fuels will eventually be depleted, many countries are focusing on the development of renewable energy sources. Therefore, there is an urgent need for high-efficiency energy storage devices to address the issues of immediacy and sustainability in renewable energy. Among the various energy storage devices, supercapacitors have garnered significant attention as electrochemical energy storage systems. They offer the advantages of a long lifespan, fast charging and discharging speeds, a wide operating temperature range, and high power density [1,2]. The current supercapacitors are still lacking in some ways, including low energy density, poor stability, and the tendency to self-discharge [3,4]. The practical applications of these limitations are limited. Improving the electrical properties of supercapacitors and achieving better capacitance characteristics can be achieved by enhancing the structure and morphology of the electrodes used. 

Graphene has a honeycomb structure in which carbon atoms are bonded via SP^2^ orbitals [5]. Graphene was first discussed in 1947 when Professor Wallace and his team used a theoretical model to calculate the energy band structure of graphite and proposed the concept of graphene [6]. It was not until 2004 that Geim and Novoselov published their results on the use of tape to physically peel off single-layer graphene [7] and reported its unique properties in journals, which attracted attention from the physics community. Until now, graphene was thought to be unstable in nature. Graphene is currently the nanomaterial with the highest specific strength. Under ideal circumstances, its Young’s modulus reaches 1.0 TPa [8]. The light transmittance of graphene is 97.7% [9], and its thermal conductivity is 5300 W/m∙K [10], both of which are higher than those of carbon nanotubes and diamond. The electron mobility of graphene is as high as 200,000 cm^2^/V∙S [11]. Such high electron mobility has great potential in electronic components [12]. Its near-perfect light transmittance and ultra-high conductivity make it highly suitable for producing transparent conductive films [13,14] and applying them to panels [15]. The unique 2D structure of graphene and its large surface area also make it applicable in the field of sensors [16,17,18]. Typical two-dimensional graphene materials have promising prospects in various fields due to their large surface area, high conductivity, and stable chemical properties. However, their use in certain fields, such as electrochemical energy storage, has not been very satisfactory. A three-dimensional conductive circuit structure is particularly important for uniformly loading and dispersing active nanostructures. It also provides fast electron and ion transport channels for nano-active materials. In recent years, there have been numerous reports on research regarding three-dimensional (3D) graphene structures. Three-dimensional graphene with various structures and properties can be prepared using different methods. The main three-dimensional graphene structures include graphene foam [19,20,21], graphene aerogel (GA) [22,23], and layered graphene [24,25,26,27]. Using the chemical vapor deposition (CVD) method to directly grow a complete graphene network structure on a three-dimensional structural substrate can address issues that traditional two-dimensional graphene films cannot resolve. These issues include a limited reaction surface area with the electrolyte and the challenge posed by ions penetrating the interior of the electrode. The three-dimensional structure of graphene perfectly overcomes these issues. Its porous structure and excellent electrical properties provide a faster pathway for charge transmission [28,29,30], which also makes graphene more suitable for use in energy storage.

The interaction between graphene and nickel causes the formation of an interfacial dipole layer. The interface dipole layer will accelerate the catalytic metal reduction reaction and facilitate the formation of nickel hydroxide.

The interaction between graphene and metal substrates has been studied theoretically, and it essentially arises from three interactions: dispersion forces (van der Waals interactions), Pauli repulsion, and donation/anti-donation bonding.

Charge transfer between transition metals and graphene causes changes in the work function of graphene, causing the Fermi level of graphene to fluctuate up and down near the Dirac point [31,32].

Electrodes have a significant impact on the performance of capacitors. Therefore, when selecting active materials, we must consider factors such as the chemical stability of the material itself, the reversibility and reproducibility of electron transfer, charge and discharge capacity, and the capacitor’s cycle life. The three categories mentioned below are the most appropriate material for current research on electrode materials: carbon-based materials, metal oxides, and conductive polymers. This study focuses on the utilization of nickel hydroxide graphene composite structures as electrode materials for supercapacitors. Nickel hydroxide (Ni(OH)_2_) is one of the potential electrode materials that can be used for supercapacitors. Depending on the preparation conditions, two crystal phases of nickel hydroxide, α and β, will be produced. There are ions intercalated in α-Ni(OH)_2_, and the water molecules in it enhance carrier mobility during the discharge process and rapidly replenish the depleted carriers on the reaction surface. Therefore, this -material has a higher specific capacitance value compared to β-Ni(OH)_2_. However, α-Ni(OH)_2_ undergoes aging during the charge and discharge process, resulting in a shorter cycle life and lower stability compared to β-Ni(OH)_2_. There is no ion intercalation in the β-Ni(OH)_2_ structure, leading to a slower transmission rate of the carriers and a smaller specific capacitance compared to using α-Ni(OH)_2_ as an electrode material. But its cycle life and stability are better than those of α-Ni(OH)_2_. The carrier transport rate of Ni(OH)_2_ can be further improved by adding graphene produced by chemical vapor deposition (CVD) process.

Table 1 presents a comparison of the use of various composite structures of transition metal oxides and graphene as materials for supercapacitor electrodes. It can be seen from this table that adding a graphene conductive layer to the original electrode material structure significantly increases its capacitance characteristics.

## 2. Experimental Section

### 2.1. Preparation of Nickel Hydroxide by Hydrothermal Method

Three-dimensional nickel foam (NF) was used as the electrode material in this experiment. First, the substrate was cut into 1 cm × 1 cm pieces. Then, it was sequentially immersed in acetone, isopropyl alcohol, hydrochloric acid, and deionized water for 15 min each while being vibrated with an ultrasonic vibrator. This pre-treatment process was carried out to prepare the substrate. A layer of graphene was grown on the surface of the nickel foam using chemical vapor deposition (CVD) in order to enhance the electrical properties of the electrode. The pre-treated nickel foam substrate was placed in a CVD furnace, and a vacuum pump was used to evacuate the furnace tube to create a vacuum. Hydrogen gas was introduced into the reaction chamber, and the furnace was heated to 1000 °C, with this temperature being held for 30 min. Methane was introduced as a carbon source gas and to control the timing of methane introduction to study the effect of methane introduction on the growth of graphene. The test pieces into which methane was introduced for 1 min, 5 min, 10 min, and 20 min were denoted as G_1_, G_5_, G_10_, and G_20_. After successfully growing CVD graphene on the surface of the 3D nickel foam substrate, nickel hydroxide was directly grown on the graphene/nickel foam substrate using a simple one-step hydrothermal method. The initial reactants for hydrothermal growth of nickel hydroxide were nickel nitrate (Ni(NO_3_)_2_·6H_2_O, 0.6543 g, 2.25 mmol) and urea (0.270 g, 4.5 mmol). They were mixed thoroughly with 45 mL of deionized water. A light-green, clear aqueous solution was produced. This mixed solution and the graphene/nickel foam substrate were placed in a 75 mL hydrothermal autoclave, heated to 200 °C, and maintained under these conditions for 6 h. According to previous research [36], the crystallinity of nickel hydroxide is optimized at a cooling rate of 10 °C/hour until reaching room temperature. Therefore, the cooling rate after the completion of hydrothermal growth of nickel hydroxide was fixed at 10 °C/hour in this study. The test pieces with a 3D-Ni(OH)_2_/NF structure, without a CVD-grown graphene layer, and with a CVD-grown graphene layer as the middle layer were named N_10_, N_10_-G_5_, and N_10_-G_10_, respectively. The sample names are shown in Table 2.

### 2.2. Material Characteristics Analysis

A D8 Advance ECO X-ray diffractometer was used to analyze the crystal structure of nickel hydroxide. A HITACHI SU8000 High-Resolution Scanning Electron Microscope (HRSEM, Hitachi High-Technologies Corporation, Tokyo, Japan) was used to observe the surface morphology of the test pieces.

### 2.3. Electrochemical Characteristics Analysis

In this experiment, we utilized a three-electrode system to measure the properties of the electrode materials that were prepared. A 1 M KOH aqueous solution was used as the electrolyte. A 1 cm × 1 cm platinum sheet was used as the counter electrode. The reference electrode was an Ag/AgCl electrode in saturated KCl. The working electrodes used in this experiment were β-Ni(OH)_2_/3D-Nickel foam and β-Ni(OH)_2_/graphene/3D-Nickel foam, which we prepared. An Admiral Squidstat Solo electrochemical instrument was used to perform cyclic voltammetry (CV), determine galvanostatic charge–discharge (GCD), and conduct electrochemical impedance spectroscopy (EIS) in order to analyze the electrochemical properties of electrode materials.

The specific capacitance value of the working electrode material prepared in this study can be calculated by integrating the area under the CV curve and incorporating it into the following formula [37]:

(1) $$C_{s}=\frac{\int idV}{2m(\Delta V)(V_{s})}$$

In the equation above, C_s_ represents the specific capacitance value (F/g), ∫idV represents the integral value of the curve, m represents the mass of the electrode material (g), ΔV represents the charge and discharge potential window (V), and Vs represents the scan rate (V/s).

The specific capacitance value of the working electrode material can be calculated by incorporating the GCD analysis results into the following formula [36]:

(2) $$C_{s}=\frac{\left(\Delta t)(I\right)}{m(\Delta V)}$$

Above, Δt represents the total discharge time (s), I represents the charge and discharge current (A), m represents the mass of the electrode material (g), and ΔV represents the charge and discharge potential window (V).

## 3. Result and Discussion

### Crystal Structure Analysis of Nickel Hydroxide

**Figure 1:**
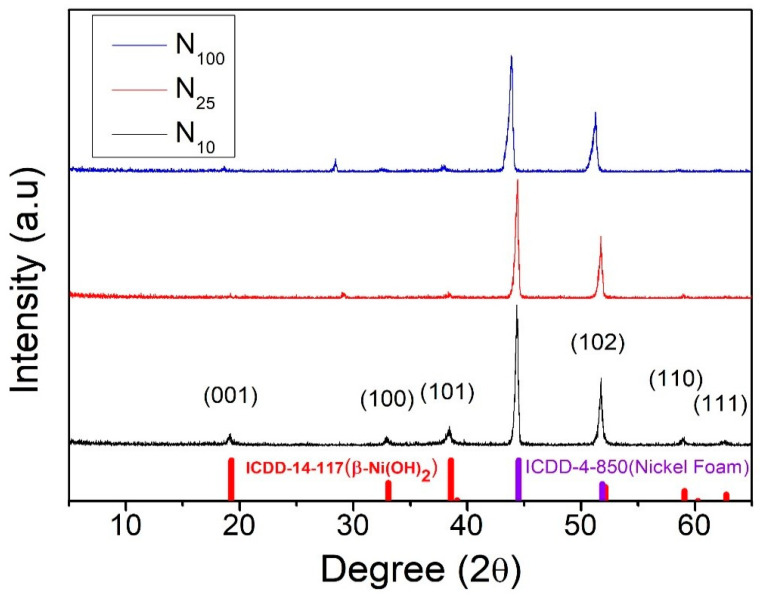
XRD patterns of β-nickel hydroxide cooled to room temperature at different rates [36].

In our previous study [36], we cooled nickel hydroxide to room temperature at three different cooling rates after hydrothermal growth in order to investigate the impact of the cooling rate on the crystallization of nickel hydroxide. Figure 1 shows the XRD patterns of N_100_, N_25_, and N_10_. The two most obvious characteristic peaks, located at 44.50° and 51.84°, are the signals of the foamed nickel substrate. The diffraction peaks located at 19.25°, 33.06°, 38.54°, 52.10°, 59.05°, and 62.72° correspond to the (001), (100), (101), (102), (110), and (111) crystal planes, respectively, of the crystallized β-phase nickel hydroxide (JCPDS 14-0117). From this result, it was determined that the phase state of nickel hydroxide synthesized via the hydrothermal method in this experiment was β-Ni(OH)_2_. In addition, the intensity of the diffraction peak became more pronounced as the cooling rate slowed down. This suggests that a slower cooling rate allows for more diffusion time, enabling the constituent atoms to arrange themselves into a more optimally structured nickel hydroxide crystal. Nickel hydroxide therefore has better crystallization properties. The quality of an electrode material’s crystallization directly affects the efficiency of the redox reactions during a capacitor’s charging and discharging processes. This helps increase the specific capacitance value of a supercapacitor.

**Figure 2:**
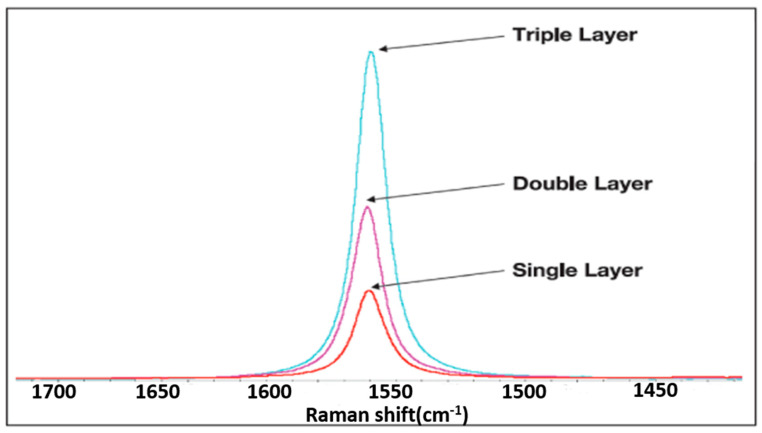
The G-peak of Raman spectrum of CVD-graphene.

Raman spectroscopy was used to analyze the crystal structure of CVD graphene. Generally, there are three characteristic signals in the Raman spectrum of graphene: the D-band (located at approximately 1350 cm^−1^), the G-band (located at approximately 1582 cm^−1^), and the 2D-band (located at approximately 2700 cm^−1^). Among them, the D-band represents disorder in the carbon materials, which is contributed by the A_1g_ breathing vibration mode. The main sources of the D-band are defects, amorphous carbon, and graphene edges. The G-band is contributed by the E_2g_ stretching mode of the sp^2^ bond of carbon atoms [38,39]. There are two methods for determining the number of graphene layers in a device. The first method is to determine the number of graphene layers based on the shape and intensity of the G-band (shown in Figure 2). The intensity of the G-band is directly related to the number of graphene layers. In addition, the number of graphene layers can also be determined according to the intensity ratio I_2D_/I_G_ of the G-band and 2D band. When the ratio is greater than 1.3, the graphene film is in a single-layer form. When the ratio between 1 and 1.3, it is double-layer graphene. A ratio between 1 and 0.5 corresponds to three-layer graphene, while graphene with more than five layers has a ratio below 0.5.

**Figure 3:**
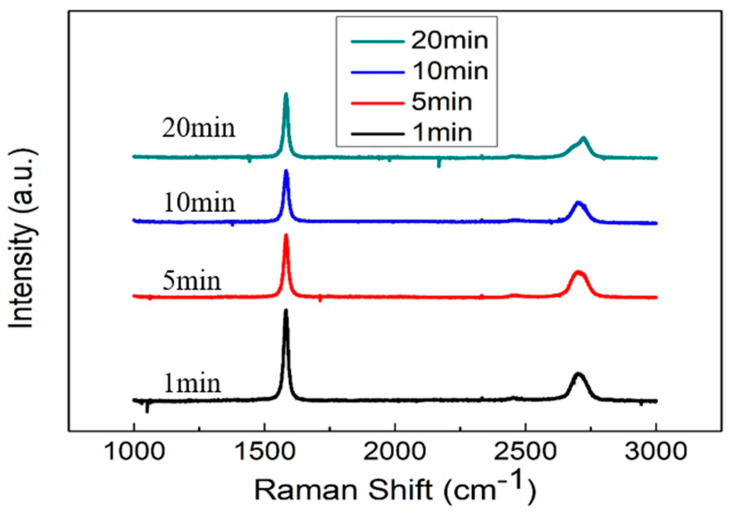
Raman spectra of CVD graphene films produced by introducing methane gas for different periods.

In this experiment, we investigated the impact of the timing of the introduction of methane gas, as a carbon source, during the growth process of CVD-grown graphene film on its crystallinity. Figure 3 shows the results of Raman spectral analysis for graphene films grown using methane for different durations (1, 5, 10, and 20 min) via the CVD process. It can be seen from Figure 3 that the G-peak intensities of the samples are 1675, 1130, 632, and 1155, respectively. From this result, it can be gleaned that the graphene (G_10_) deposited by introducing methane for 10 min has the fewest layers and the lowest thickness, followed by the graphene (G_5_) deposited by introducing methane for 5 min. After analysis and calculation, it was found that the I_2D_/I_G_ ratios of the samples, in which methane was introduced for 1, 5, 10, and 20 min, were 0.369, 0.450, 0.559, and 0.412, respectively. From this result, it can be gleaned that the graphene (G_10_) layers deposited by methane for 10 min are the smallest in number and the thinnest, followed by those for the 5 min deposition (G_5_). This trend is consistent with our analysis of the G-peak intensity results. In subsequent experiments, the composite electrode structures of Ni(OH)_2_/G_10_/NF and Ni(OH)_2_/G_5_/NF were compared to study the effect of the number of graphene layers (G_10_ and G_5_) on the characteristics of supercapacitors.

**Figure 4:**
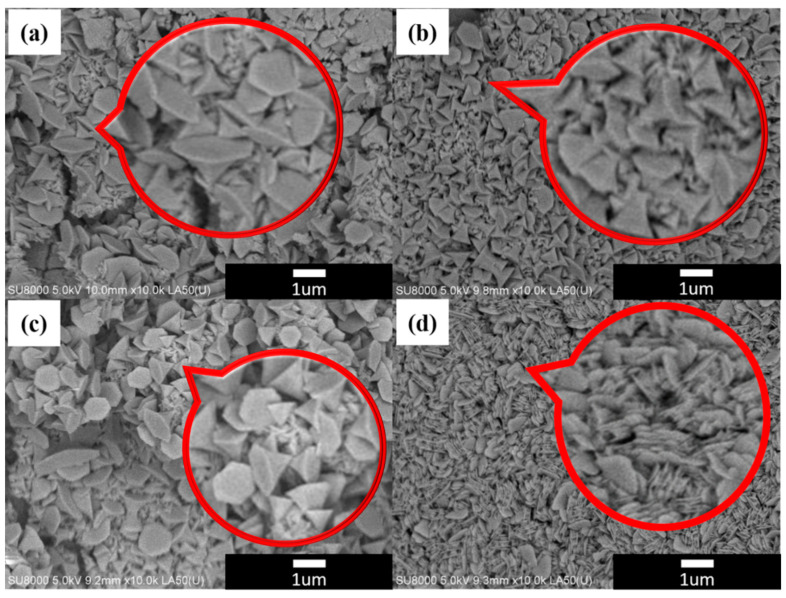
SEM images of nickel hydroxide with different structures: (**a**) N_100_; (**b**) N_25_; (**c**) N_10_; and (**d**) N_10_-G_10_.

In this experiment, HR-SEM was used to observe the surface micromorphology of nickel hydroxide materials, as shown in Figure 4. It can be observed in Figure 4a–c that the nickel hydroxide films grown at different cooling rates after the hydrothermal process are composed of cone-shaped crystals with sizes ranging from 500 nm to 1 μm. Furthermore, there was not much of a change in morphology. It can be observed in Figure 4d that after CVD graphene was grown on the nickel foam substrate, the surface activation energy and morphology of the graphene and pure nickel foam changed, resulting in different morphologies of the hydrothermally grown nickel hydroxide. The morphology of the hydrothermally grown nickel hydroxide film changed, consisting of vertically and intricately grown flaky nickel hydroxide on the surface of the test piece. This structural change increased the overall surface area of the test piece (electrode). The increased surface area of the working electrode facilitates its contact with OH^-^ ions in the electrolyte and promotes oxidation–reduction reactions, thereby enhancing its capacitive performance. Therefore, combining a graphene conductive layer with nickel hydroxide to form a composite structural material offers great potential advantages with regard to designing an electrode material for supercapacitors.

**Figure 5:**
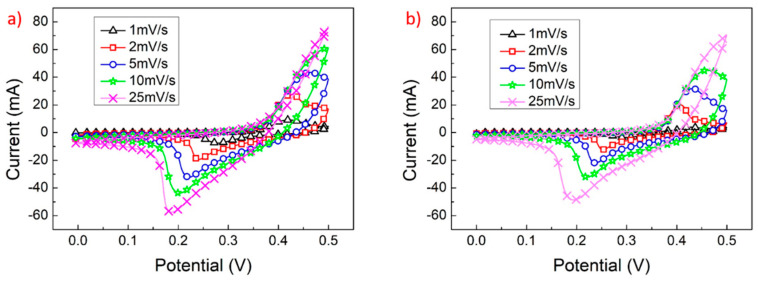
CV curves of (**a**) N_10_-G_5_ and (**b**) N_10_-G_10_.

**Figure 6:**
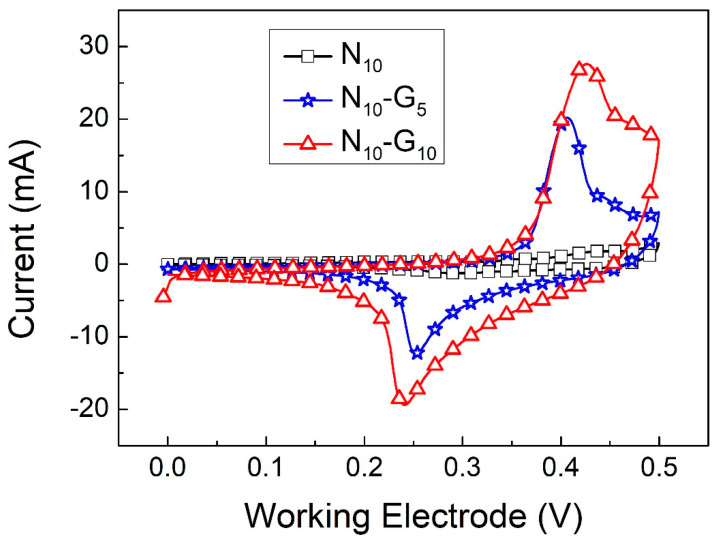
CV curves of N_10_, N_10_-G_5_, and N_10_-G_10_ at a scan rate of 2 mV/s.

In this experiment, we also investigated the impact of incorporating G_5_ and G_10_ graphene layers between the nickel hydroxide and nickel foam working electrode materials on the capacitance properties of the supercapacitor. Figure 5a,b show the CV curves of N_10_-G_5_ and N_10_-G_10_ at scan rates ranging from 1 mV/s to 25 mV/s. Figure 6 shows the CV curves of N_10_, N_10_-G_5_, and N_10_-G_10_ at a scan rate of 2 mV/s. Calculated using Formula (1), the specific capacitance values of the working electrodes using three different materials are 378.9, 612.8, and 843.1 F/g, respectively. After adding the graphene conductive intermediate layer, the capacitance value of the supercapacitor significantly improved. The redox stability of the electrode can also be determined according to the ratio of the oxidation peak current (I_pb_) to the reduction peak current (I_pa_). The I_pb_/I_pa_ values of N_10_, N_10_-G_5_, and N_10_-G_10_ are 0.61, 0.61, and 0.70, respectively. Additionally, the graphic symmetry of N_10_-G_10_ is superior to that of N_10_ and N_10_-G_5_, indicating better redox stability. This is because the G_10_ interlayer has fewer layers and a lower thickness, improving its conductivity compared to the G_5_ interlayer. This acceleration of charge transfer during the redox process enhances the stability of the redox reaction.

**Figure 7:**
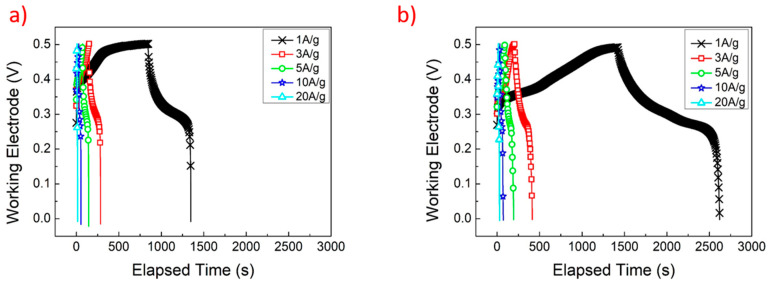
The GCD curves of (**a**) N_10_-G_5_ and (**b**) N_10_-G_10_.

**Figure 8:**
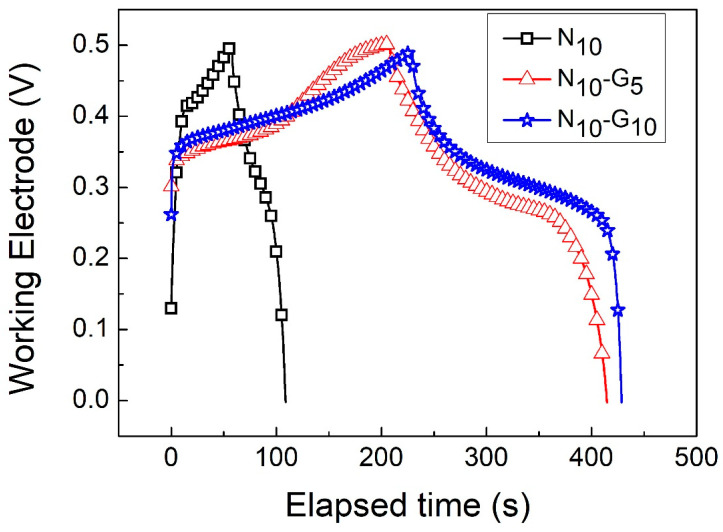
GCD curves of N_10_, N_10_-G_5_, and N_10_-G_10_ at 3 A/g current density.

Figure 7a,b show the GCD curves of N_10_-G_5_ and N_10_-G_10_ at current densities ranging from 1 A/g to 20 A/g. Figure 8 shows the GCD curves of N_10_, N_10_-G_5_, and N_10_-G_10_ at 3 A/g. Using Formula (2), the specific capacitance values of N_10_, N_10_-G_5_, and N_10_-G_10_ could be calculated. The results are shown in Table 3. At a current density of 3 A/g, the specific capacitance values of N_10_, N_10_-G_5_, and N_10_-G_10_ are 538.7, 1095.2, and 1218.9 F/g, respectively. The specific capacitance value of N_10_-G_10_ was found to be increased by 126% compared to that of N_10_. It can be inferred that by inserting an intermediate layer, such as a highly conductive CVD graphene layer, between the hydrothermally grown nickel hydroxide nanostructure and the 3D nickel foam substrate as a working electrode, the capacitance characteristics of a supercapacitor can be improved.

The energy efficiency of a capacitor is an important parameter that can be calculated using the following formula [40]:

(3) $$Energy\;efficiency=\frac{energy\;density(discharge)}{energy\;density(charge)}\times 100$$

The energy efficiency of supercapacitors incorporating electrode materials with varying structures can be calculated using Formula (3). The results are summarized in Table 4, as shown below.

We compiled some research results related to our study in Table 5.

**Figure 9:**
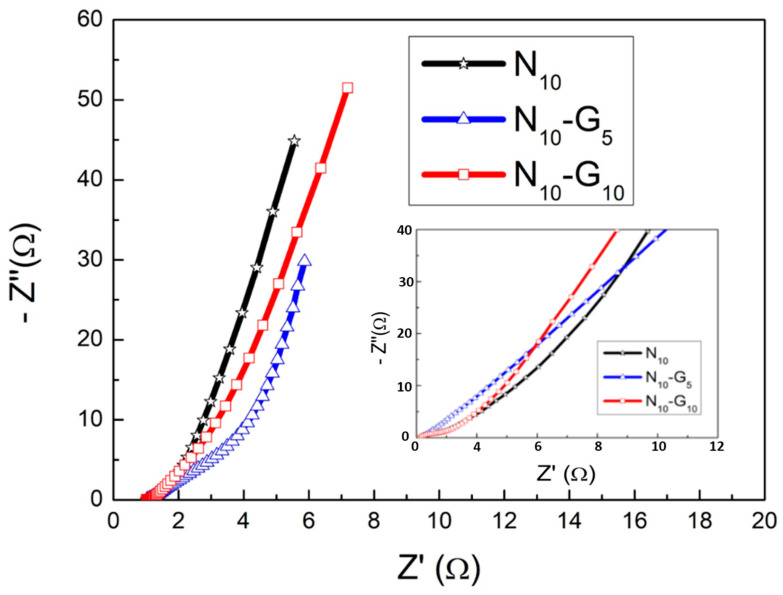
EIS Nyquist diagram of the performance of test pieces N_10_, N_10_-G_5_, and N_10_-G_10_ as electrode materials.

Figure 9 shows the Nyquist diagrams of N_10_, N_10_-G_5_, and N_10_-G_10_ as tested via EIS.

This graph displays the AC impedance at various AC frequencies, separated into the real part (Z′) and the imaginary part (-Z″). The X value of the curve that intersects with the X-axis in the high-frequency area represents the ESR (equivalent series resistance). This resistance is a combination of the solution impedance and the internal resistance of an electrode. A typical Nyquist diagram will have a semicircle in the high-frequency region. The diameter of this semicircle corresponds to R_ct_ (charge transfer resistance). This resistance value can serve as a reference for the speed of charge transfer within the electrode. The smaller the R_ct_ value, the faster the charge transfer rate inside the electrode [46]. It is known from the literature [47] that the EIS Nyquist plot of supercapacitors falls between that of typical battery materials and ideal capacitor materials. It is not easy to see a clear semicircle in the plot. Therefore, in this experiment, we utilized simulation software to estimate ESR and R_ct_ values by selecting multiple points on the bending line in the high-frequency region. Calculated from the intercept of the X-axis in the figure, the equivalent series resistances (ESRs) of the three test pieces are very close, all being approximately 1.01 Ω, while the charge transfer resistances (R_cts_) are 0.22 Ω, 0.15 Ω, and 0.09 Ω, respectively. As a result of hydrothermal growth of nickel hydroxide, the cooling rate slowed down, resulting in a decrease in the charge transfer resistance inside the electrode, as suggested by these results. In other words, the charge transfer resistance inside the electrode of specimen N_10_-G_10_ was the smallest, indicating that the charge transfer process was more efficient. From the previous Raman spectrum analysis, it is known that G_10_ has fewer layers of graphene. It can be inferred that graphene with fewer layers has excellent electrical conductivity, making it suitable for β-Ni(OH)_2_/CVD-Graphene/3-D-NF (nickel foam). The composite structure, serving as the electrode of the supercapacitor, makes a positive contribution to enhancing the specific capacitance value of the entire capacitor. 

**Figure 10:**
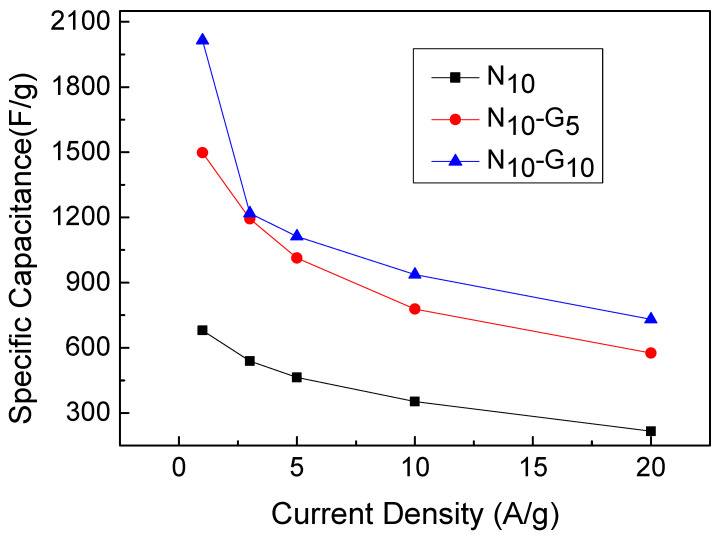
Ratio capacitance values of N_10_, N_10_-G_5_, and N_10_-G_10_ changing with current density.

Figure 10 shows a graph depicting the variation in the specific capacitance values of N_10_, N_10_-G_5_, and N_10_-G_10_ with respect to the charge and discharge current density. It can be observed that the specific capacitance values of the test samples with the added graphene conductive layer are significantly higher compared to those without this layer. Upon comparing the data regarding N_10_-G_10_ and N_10_-G_5_, it can be observed that the specific capacitance values of the two are actually quite similar when charging and discharging at low currents. However, when charging and discharging under high-current conditions of 20 A/g, the specific capacitance values of N_100_, N_25_, N_10_, N_10_-G_5_, and N_10_-G_10_ are 171.4, 208.1, 216.3, 547.6, and 730.6 F/g, respectively. Compared to the values measured at a current density of 3A/g, these data reveal that the original specific capacitance values of 38.4%, 47.8%, 40.1%, 50.0%, and 60.0% were maintained. Among these test pieces, N_10_-G_10_ can still retain 60% of its specific capacitance value after being charged and discharged at a high current density of 20 A/g. This indicates that it has a superior ability to withstand high-current charging and discharging. The graphene conductive layer introduced into the composite electrode structure significantly improved the device’s high-current charge and discharge characteristics.

**Figure 11:**
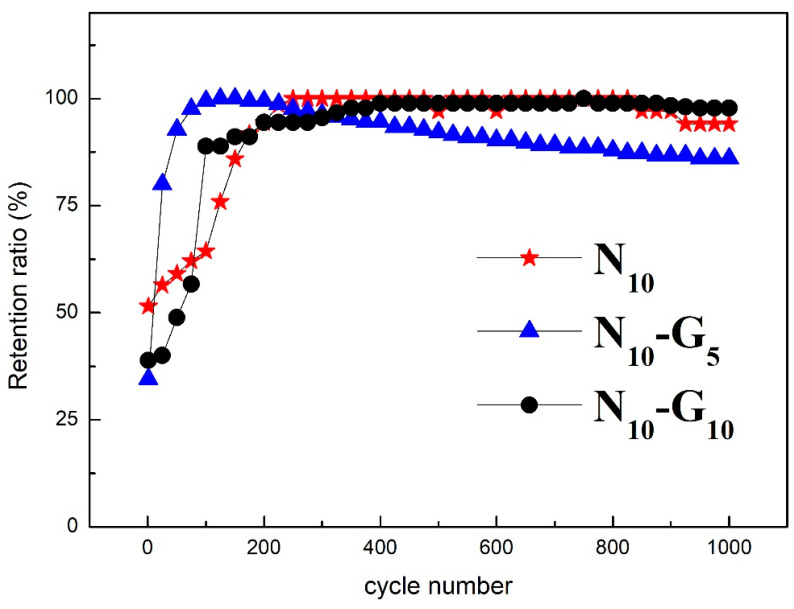
Changes in specific capacitance values of N_10_, N_10_-G_5_, and N_10_-G_10_ after 1000 consecutive charge and discharge cycles.

Figure 11 shows the changes in specific capacitance values of N_10_, N_10_-G_5_, and N_10_-G_10_ after 1000 consecutive charge and discharge cycles at a current density of 20 A/g.

Figure 11 shows the changes in the specific capacitance values of N_10_, N_10_-G_5_, and N_10_-G_10_ after 1000 consecutive charge and discharge cycles at a charge and discharge current density of 20 A/g. From the figure, it can be concluded that after 200 cycles, the specific capacitance value for N_10_-G_5_ and N_10_-G_10_ rapidly increases to the maximum specific capacitance value. This phenomenon is due to the improved wetting effect between the electrode material and the electrolyte. After a charge-discharge cycle, the electrolyte will penetrate the electrode’s interior more easily, allowing for a more thorough infiltration. This facilitates the entry of ions from the electrolyte into the electrode’s interior. Therefore, the specific capacitance value will rise sharply to its maximum value. After 1000 charge and discharge cycles, the specific capacitance values of N_10_, N_10_-G_5_, and N_10_-G_10_ remain 94.1%, 86.1%, and 99.2%, respectively. Notably, N_10_-G_10_ exhibits an impressive specific capacitance retention of up to 99.2%. Such an excellent cycle life proves that this composite structural material is highly reliable as an electrode material for supercapacitors.

## 4. Conclusions

In this study, we successfully incorporated a double- to triple-layer graphene highly conductive layer between hydrothermally grown β-nickel hydroxide and a 3D-foamed nickel substrate using the CVD method. When the microscopic morphology of β-nickel hydroxide grown on graphene changes from a cone shape to a lamellar structure, it can give rise to a larger specific surface area. This change is beneficial for increasing the specific capacitance value of a capacitor. The β-nickel hydroxide prepared through hydrothermal synthesis was cooled to room temperature at various rates, significantly affecting the capacitance characteristics. The research results reveal that the capacitance characteristics of the N_10_ specimen are the best among the three samples with different cooling rates. Under the condition of a current density of 3 A/g, the capacitor was subjected to constant current charging and discharging experiments. The capacitor had a specific capacitance value of 538.7 F/g. Among the conductive layers with CVD graphene added as a composite electrode structure, the N_10_-G_10_ test piece exhibited the highest specific capacitance value, amounting to 1218.9 F/g. This represents a 126% increase in specific capacitance compared to the sample without graphene. It still retained 99.2% of its specific capacitance value after 1000 consecutive charge and discharge cycles. The β-Ni(OH)_2_/CVD-Graphene/3D-Nickel Foam electrode material prepared in this study exhibited an excellent specific capacitance and cycle life. Furthermore, its production process is fast and simple. It has the potential to be applied to future supercapacitors and various energy storage components.

## Figures and Tables

**Table 1:** Comparison of the capacitances obtained when using different transition metal oxide and graphene composite structures as supercapacitor electrode materials.

Electrode Structure	Charge and Discharge Current (A/g)	Capacitance without Graphene (F/g)	Capacitance with Graphene (F/g)	Reference in This Work
PPy/Multilayer Graphene/Cu NPs	1	214	845	[33]
NiFe/Graphene	1	264	845	[34]
Na_0.11_MnO_2_/3DG	0.2	686	1240	[35]
β-Ni(OH)_2_/graphene	1	538.7	2015.2	This work

**Table 2:** The names given to the samples in this study.

Cooling Rate (Sample Structure)	10 °C/hour, (β-Ni(OH)_2_/NF)	10 °C/hour, G_5_ Middle Layer (β-Ni(OH)_2_/G_5_/NF)	10 °C/hour, G_10_ Middle Layer (β-Ni(OH)_2_/G_10_/NF)
sample name	N_10_	N_10_-G_5_	N_10_-G_10_

**Table 3:** The charging and discharging specific capacitance values of N_10_, N_10_-G_5_, and N_10_-G_10_ at different current densities.

Current Density (A/g)	N_10_	N_10_-G_5_	N_10_-G_10_
1	680	1498	2015
3	539	1193	1219
5	464	1013	1112
10	353	778	937
20	216	576	731

**Table 4:** Energy efficiency of supercapacitors using electrode materials with different structures employed in this study.

Sample Number	N_10_	N_10_-G_5_	N_10_-G_10_
Energy efficiency	90.4%	65%	78%

**Table 5:** A comparison between recent studies using different materials as supercapacitor electrode materials and the results of this article.

Materials	Specific Capacitance (F/g)	Test Parameter (CV/GCD)	Ref. in This Work
MoS_2_ nanoworms	1866	5 mV/s (CV)	[41]
MgCo_2_O_4_ nanosheets	1460	2 mA/cm^2^ (GCD)	[42]
PANI/Graphene/MIL-100(Fe)	638	1 A/g	[43]
rGO/ZnCoS	891	1 A/g	[44]
(Co_x_Ni_1-x_)_2_P/Co_x_Ni_1-x_S	1561	1 A/g	[45]
Ni(OH)_2_/Graphene	2015	1 A/g	This work

## Data Availability

The data presented in this study are available on request from the corresponding author.
